# Collaborating Across Organizational Boundaries to Develop, Evaluate, and Implement eHealth: Scoping Review

**DOI:** 10.2196/67839

**Published:** 2025-06-10

**Authors:** Aafke G Coopmans, Remco S Mannak, Anna M Braspenning, Eveline J M Wouters, Inge M B Bongers

**Affiliations:** 1 Tranzo Tilburg School of Social and Behavioral Sciences Tilburg University Tilburg The Netherlands; 2 Department of Organization Studies Tilburg School of Social and Behavioral Sciences Tilburg University Tilburg The Netherlands; 3 School of Allied Health Professions Fontys University of Applied Sciences Eindhoven The Netherlands

**Keywords:** eHealth, digital health, development, evaluation, implementation, interorganizational collaboration, organizational boundaries, dialogical learning mechanisms, boundary spanning

## Abstract

**Background:**

The success of eHealth relies on interorganizational collaboration (IOC) throughout the development, evaluation, and implementation phases of eHealth deployment. This IOC is complex, as it involves a diversity of organizations from different sectors, such as technological, academic, health care, and governmental organizations, collaborating to deploy eHealth. Between these organizations, organizational boundaries, defined as the demarcation of an organization from its environment, arise. When these boundaries are perceived as aligned and enable complementarity, IOC is facilitated. By contrast, misalignment of organizational boundaries can hinder IOC. A dialogical learning mechanism, defined as a learning process that occurs when boundaries hinder IOC, can support learning how to navigate such boundaries. However, it is difficult to determine whether and when organizational boundaries facilitate or hinder IOC, and which dialogical learning mechanisms can be used to address these challenges during eHealth deployment. Previous literature presents the barriers and facilitators of IOC during eHealth deployment only for subsets of organizations or specific phases, leaving their generic versus phase specific applicability uncharted.

**Objective:**

This scoping review aims to identify whether, and under what circumstances, organizational boundaries facilitate or hinder IOC during the development, evaluation, and implementation of eHealth.

**Methods:**

A scoping review was conducted using searches in the PubMed, PsycINFO, CINAHL, and Web of Science databases. Articles were eligible for inclusion if they were empirical studies written in English or Dutch and contained findings on factors influencing IOC during the development, evaluation, or implementation phases of eHealth deployment. The search yielded 11,867 articles, of which 16 met the inclusion criteria. Open and axial coding of the extracted findings was performed to identify organizational boundaries and dialogical learning mechanisms that hindered or facilitated IOC during eHealth deployment.

**Results:**

In each phase, different organizational boundaries either hindered or facilitated IOC. The dialogical learning mechanism identification was crucial for enhancing IOC and was supported by training or by establishing IOC from previous relationships. Additionally, the learning mechanism coordination improved IOC and depended on the involvement of boundary spanners (ie, individuals who span organizational boundaries) and the use of boundary objects (ie, objects which help bridge different social worlds). Furthermore, the mechanism reflection, fostered through open and frequent communication, facilitated IOC. The dialogical learning mechanism transformation did not influence IOC during any phase of eHealth deployment.

**Conclusions:**

IOC in eHealth deployment is a dynamic process that depends on the dialogical learning mechanisms identification, coordination, and reflection to navigate organizational boundaries. This review is the first to present organizational boundaries and dialogical learning mechanisms that influence IOC across the different phases of eHealth deployment. However, further research that explicitly considers these phases is needed to deepen the understanding of IOC in eHealth deployment.

## Introduction

eHealth, the use of information and communication technologies to support health and health care [1], is revolutionizing the field. It enables the provision of care independent of time and place [2] and offers novel treatment options. Examples are the use of virtual reality to reduce pain and anxiety in children undergoing medical treatment [3], and the use of wearables to continuously monitor symptoms [4]. Because of these possibilities, eHealth is advocated as a means of addressing several challenges faced by health care, such as the high demand for care [5], rising health care costs [6], and the extensive workload of health care staff [7]. However, the envisioned benefits are not fully realized when eHealth is implemented in practice [8]. Its implementation is challenged by technological, financial, organizational, and legal aspects [9-11]. Improving this implementation requires interorganizational collaboration (IOC), in which organizations engage in an iterative cycle of development, evaluation, and implementation phases [10,12,13]. Through IOC, organizations can complement each other’s expertise and jointly develop a comprehensive understanding of user needs, health care requirements, and suitable technology that addresses those needs and wants [12]. In particular, IOC across the development, evaluation, and implementation phases is crucial for the success of eHealth in subsequent stages [12]. For example, technological features that can enhance successful implementation must be addressed during the development phase.

IOC, however, has proven to be rather complex. Although the health care literature has identified multiple facilitators of IOC, such as mutual trust, management, and communication [14-16], it has also reported several barriers that contribute to its complexity [14-16]. These barriers, including the lack of a clear goal and power imbalances, hinder IOC and pose a threat to collaboration outcomes in health care [14-16]. A subset of these barriers and facilitators can be explained by the presence of organizational boundaries, defined as “the demarcation of an organization from its environment” [17]. Each organization operates with its own goals, values, priorities, and resources, which gives rise to these boundaries. These organizational boundaries can either facilitate or hinder IOC. On the one hand, when boundaries are aligned and enable complementarity, IOC is supported. For example, complementary knowledge and skills [18], along with aligned expectations, facilitate IOC in health care [14,19]. On the other hand, when boundaries are misaligned or foster competition, they can lead to conflicts [20] and tensions [16,21] that hinder IOC. For instance, misaligned goals and priorities can impede IOC [22]. In addition, differences in work culture or competing organizational identities may hinder collaboration [22,23]. As these boundaries can obstruct IOC, learning how to navigate them is crucial to addressing its complexity.

Learning to deal with boundaries can be achieved through a dialogical learning mechanism, defined as a learning process that occurs when a boundary is encountered [24]. Specifically, the 4 dialogical learning mechanisms proposed by Akkerman and Bakker [24,25], namely, “identification,” “coordination,” “reflection,” and “transformation,” focus on learning from boundaries that result in a discontinuation of action or interaction [24,25]. The identification mechanism initiates the learning process, in which a person, group, or organization questions its own identity by comparing it with the identity of others. This process leads to renewed insight into one’s own identity, the identity of the other, and the boundary between them. After this, a process of coordination begins, focusing on searching for or developing procedures and methods that enable exchanges across boundaries [24]. For example, individuals (boundary spanners) [26] or objects (boundary objects) [27] situated between 2 worlds can be used to bring persons, groups, or organizations together. Next, the process of reflection occurs, in which learning involves defining one’s own perspective and gaining insight into the perspectives of others through perspective taking, where an exchange of viewpoints takes place [24]. Additionally, a process of transformation may occur, in which awareness of a shared problem and IOC leads to the formation of new identities and practices [24]. All 4 learning processes can contribute to understanding how to navigate boundaries in diverse contexts. Moreover, individuals, groups, or organizations can use these dialogical learning mechanisms to optimize IOC [28].

Knowing whether and when organizational boundaries facilitate or hinder IOC, and which dialogical learning mechanisms can be used to optimize IOC, is particularly relevant for eHealth deployment. To deploy eHealth, technological, academic, health care, and governmental organizations work together [29-31]. During this IOC, organizations may encounter organizational boundaries [32,33], which can arise within and across the development, evaluation, and implementation phases. To prevent these boundaries from hindering IOC, boundary spanning (eg, activities, practices, and processes across boundaries) is required [26]. However, whether and when organizational boundaries hinder or facilitate IOC, and which dialogical learning mechanisms can be used to address these boundaries, are difficult to determine from the literature. Studies on the barriers and facilitators of IOC often focus on dyads, defined as IOC between 2 organizations. For example, some studies examine IOC between academic and industry organizations [34,35], while others explore IOC between academic and community organizations [36]. Additionally, other studies focus on IOC during the development phase [18], the evaluation phase [37], the implementation phase [38], or across multiple phases [21]. Because of this fragmentation across different dyads and phases, it remains unclear whether a boundary or a learning mechanism is specific to a particular dyad or phase, or applicable across all. Therefore, this scoping review aims to identify whether and when organizational boundaries facilitate or hinder IOC during eHealth development, evaluation, and implementation. The hypothesis is that applying a learning mechanism can help transform an organizational boundary from hindering to facilitating IOC within a given phase or dyad.

## Methods

### Study Design

A scoping review was conducted by a multidisciplinary team of collaboration and eHealth researchers. The PRISMA-ScR (Preferred Reporting Items for Systematic Reviews and Meta-Analyses Extension for Scoping Reviews; Multimedia Appendix 1) checklist was used to ensure that all relevant items were reported [39]. The review protocol was not preregistered.

### Eligibility Criteria

Studies were eligible for inclusion if they met the following criteria: (1) written in English or Dutch, (2) peer reviewed, (3) empirical studies, (4) containing findings on IOC, and (5) covering (parts of) the development, evaluation, or implementation phases of eHealth. There were no restrictions on the publication date due to the explorative nature of this scoping review. Table 1 presents the specific definitions and operationalizations of the eligibility criteria.

**Table 1:** Eligibility criteria used to screen search results on eligibility.

Criteria	Eligibility criteria
Language	Written in English or Dutch
Publication	Peer-reviewed scientific articles
Study design	Empirical studies: studies in which the authors collected their own data
Findings	Containing findings on what is influencing collaboration between 2 or more organizations
eHealth	Covering (parts of) the development^a^, evaluation^b^ and implementation^c^ of eHealth^d^

^a^Refers to developing (prototypes of) technology according to its intended purpose [2].

^b^Refers to evaluating the effectiveness of eHealth in its intended context [2].

^c^Refers to the adoption, dissemination, and long-term use of eHealth in its context [2].

^d^Refers to using information and communication technologies to support health and health care [1].

### Search Strategy

Searches were conducted in PubMed, PsycINFO, CINAHL, and Web of Science, including articles published up to January 23, 2024. AGC developed a search strategy using terms related to organizations, combined with IOC and eHealth, using Boolean operators. An example of a search string used in Web of Science is as follows: ((AB=(interorgani* OR inter-organi* OR alliance OR consorti* OR network* OR inter-firm OR interfirm OR “joint venture” OR joint-venture OR coalition OR organization* OR organisation* OR firm* OR compan* OR stakeholder* OR public OR private OR academic OR industr* OR universit*)) AND AB=(collaborat* OR cooperat* OR relationship* OR partnership*)) AND (AB=(“digital health” OR “electronic health” OR “mobile health” OR “web-based health” OR eHealth OR e-Health OR mHealth OR m-health OR telemedicine OR tele-medicine OR telehealth OR tele-health OR “eMental health” OR “e-Mental health”). Multimedia Appendix 2 provides an overview of all search strings.

### Study Selection

AGC screened the titles and abstracts of these articles for eligibility using the criteria shown in Table 1. Simultaneously, AMB screened a subset of the titles and abstracts to examine the interrater reliability (Cohen κ) and advance the eligibility criteria. Once the screening of the first subset was completed, conflicts in inclusion were discussed iteratively. Following this, a discussion was held with RSM, EJMW, and IMBB to refine the eligibility criteria. Next, AGC screened the remaining titles and abstracts. Finally, AGC screened the full texts and, in case of any doubts, AGC consulted RSM, AMB, EJMW, and IMBB.

### Data Extraction and Data Analysis

AGC performed the data extraction and analysis. First, AGC extracted the general study characteristics of the articles, such as the title, authors, year, study aim, and study design, into a data extraction form. This form was an Excel spreadsheet (Microsoft Corporation) with multiple subtabs and was pilot tested by RSM, EJMW, and IMBB to ensure it covered the correct data categories. In addition, AGC extracted the phase, sectors, organizations, disciplines, and findings into the form. Second, AGC applied open and axial coding to the findings to identify the barriers and facilitators of IOC. The barriers and facilitators were grouped into organizational boundaries and the 4 dialogical learning mechanisms using the definitions in Table 2. This was followed by categorizing them into the deployment phases, using the definitions in Table 1. AGC iteratively refined the data extraction and codes to structure the findings. Additionally, discussions between AGC, RSM, EJMW, and IMBB were held to reach a consensus on the occurrence of organizational boundaries and dialogical learning mechanisms throughout eHealth deployment. AGC did not conduct a critical appraisal of each article but selected all articles from high-quality databases. This aligns with the aims of scoping reviews, which focus on exploring the extent, range, or nature of the literature rather than its quality [39]. Furthermore, no generative artificial intelligence was used during data extraction and analysis, although AGC used Grammarly (Grammarly Inc.) to improve sentences while rewriting and editing the manuscript.

**Table 2:** Definitions of an organizational boundary and dialogical learning mechanisms used for coding.

Concept	Used definition
Organizational boundary	The demarcation between the organization and its environment [17].
Identification	A renewed insight into how different practices or individuals differ from each other [24].
Coordination	The development of new or usage tools and procedures to enhance collaboration [24].
Reflection	Identification of boundaries and perspective making [24].
Transformation	The development of new practices or identities caused by interorganizational collaboration [24].

## Results

### Study Selection

**Figure 1:**
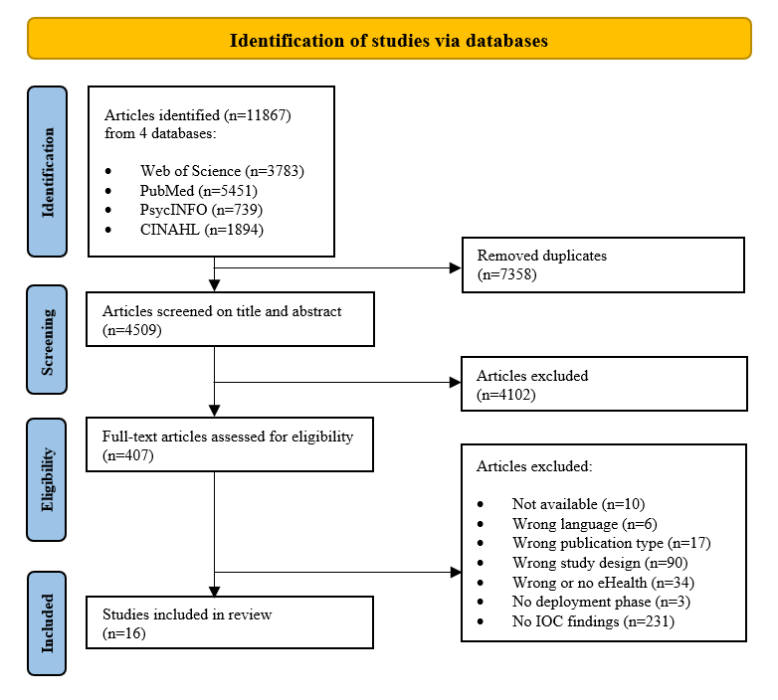
PRISMA (Preferred Reporting Items for Systematic Reviews and Meta-Analyses) flow chart. IOC: interorganizational collaboration.

Figure 1 illustrates the study selection process, which began with search results that yielded 11,867 articles and resulted in the inclusion of 16 articles. Eliminating duplicates from the search results identified 4509 (38%) unique articles. Agreement between AGB and AMB was reached for 308 of 359 abstracts (85.8%) regarding inclusion (n=30), exclusion (n=277), and maybe (n=1), resulting in a moderate κ score of 0.51. The primary reason for excluding an article based on its title or abstract was that the authors did not aim to study IOC or eHealth. A total of 407 articles were deemed eligible based on title and abstract and proceeded to the full-text screening phase. The primary reason for excluding an article based on the full-text assessment was the absence of explicit findings, such as barriers and facilitators, related to factors influencing IOC during the development, implementation, or evaluation phase of eHealth.

### Overview of the Eligible Literature

Table 3 provides an overview of the study characteristics of the eligible literature. It shows that exploratory studies from Western countries, published after 2010 and focusing on the implementation phase, were predominant among the eligible studies. Additionally, the study characteristics indicate that the barriers and facilitators of IOC were obtained in different ways. This is best illustrated by the study objectives. The authors of 9 articles [40-48] primarily aimed to identify the barriers and facilitators of IOC in eHealth, as stated explicitly in their objectives; for example, “Our study aims to identify the barriers and facilitators associated with academia–industry collaborations in digital health in middle- and high-income countries” [43]. Within these studies, the focus was predominantly on academic-industry and health care-academia collaborations, without emphasizing a specific deployment phase [40-47]. However, the authors of the remaining 7 articles [49-55] did not primarily aim to study IOC. Instead, they described the involvement of organizations from academia, industry, health care, and government within their projects or study samples, along with the processes or barriers and facilitators related to the development, evaluation, or implementation of eHealth; for example, an objective was “To identify implementation lessons from the United Kingdom Delivering Assisted Living Lifestyles at Scale (Dallas) program—a largescale, national technology program that aims to deliver a broad range of digital services and products to the public to promote health and wellbeing” [49]. Additionally, the authors studied governmental and hospital networks aimed at promoting eHealth [50,54]. However, the focus of these studies was not on the organizations involved in the collaborations or the factors influencing IOC. As a result, the barriers and facilitators of IOC were identified more spontaneously and less extensively compared with the studies that primarily aimed to examine IOC.

**Table 3:** Overview of eligible literature.

Study	Country	Objective	Data	Phase
Cho and Mathiassen [54]	The United States	To conduct a multilevel process analysis on the adoption of a telehealth innovation.	Documents Interviews Observations Fieldnotes	Implementation
Wakefield et al [42]	The United States	To obtain collaboration experiences of clinical access hospitals and a referral hospital collaborating on enhancing their systems.	Documents Interviews	Implementation
Devlin et al [49]	The United Kingdom	To describe the lessons learned from eHealth implementation.	Documents Interviews Observations	Implementation
Naik et al [41]	The United States	To describe partnership building between a research team and primary care provider.	Communication notes Field notes Transcribed meetings	Evaluation
Halje et al [46]	Sweden	To gain experiences from researchers and clinicians in collaborative research.	Interviews	Development and evaluation
Garmann-Johnsen and Eikebrokk [50]	Norway	Questioning the assumption that welfare technologies can be built from the bottom-up.	Literature review Interviews	Implementation
Swinkels et al [51]	The Netherlands	To present challenges of patients, health care professionals, and entrepreneurs while adopting eHealth in primary care.	Interviews Focus groups Meeting notes	Implementation
Ford et al [44]	The United States	To describe an academic-industry partnership.	Qualitative feedback	Evaluation
Liu et al [43]	The United States	To obtain the barriers and facilitators of academia-industry collaborations.	Interviews	Development and implementation
Terrill et al [40]	The United States	To describe the collaboration process between game developers and rehabilitation researchers; as well as to provide a development framework for mHealth^a^ development.	No data collection	Development
Bally and Cesuroglu [52]	The Netherlands	To understand the complex eHealth implementation environment.	Interviews Focus groups	Implementation
Austin et al [47]	The United Kingdom	To obtain the benefits, barriers, solutions, and pathways for a successful collaboration.	Email questionnaire	Development
Ford et al [45]	The United States	To explore collaborative experiences of representatives of digital health companies and academic institutions that work together.	Interviews	Development and evaluation
Moltrecht et al [55]	The United Kingdom	To present the development process.	Observations Public engagement events Knowledge synthesis Co-design workshops Participatory workshops Prototype testing sessions	Development
Benjamins et al [53]	The Netherlands	To obtain the barriers and facilitators of implementing eHealth.	System data Observations Questionnaires Focus groups	Implementation
Callens and Verhoest [48]	Belgium	To explain the collaborative innovative process of public-private partnerships.	Interviews Survey	Development

^a^mHealth: mobile health.

### Organizational Boundaries and Dialogical Learning Mechanisms Influencing IOC

#### Overview

This section discusses the organizational boundaries that either facilitated or hindered IOC in the eligible literature, as well as how this interaction varied across deployment phases and dialogical learning mechanisms. The dialogical learning mechanism transformation and the health care-industry dyad are not covered, as neither was identified in the eligible literature.

#### Organizational Boundaries

Organizational boundaries both hindered and facilitated IOC across all eHealth deployment phases. These boundaries were either generic or phase specific. The generic organizational boundaries were observed during academic-industry collaborations [43,45,47], academic-industry-health care collaborations [53], or academic-industry-health care-government collaborations [51], spanning 2 or 3 deployment phases. Specifically, organizational boundaries that hindered IOC across all phases included misaligned expectations [43,47,51] and conflicts of interest [45]. Additionally, differences in operational processes [45] and timelines [43,47] hindered IOC. By contrast, flexible organizational boundaries [45] or aligned expectations [45] facilitated IOC. Furthermore, organizational boundaries that hindered IOC in 2 phases included misaligned goals [43], conflicting priorities [47], and differing visions that led to tensions [53]. By contrast, aligned goals [43] and complementary roles [43] facilitated IOC. The differences in timelines [47] and conflicting scientific and business priorities [43] were specifically related to the academic-industry dyad. To illustrate, “Interviewees from both academia and industry identified timeline as a barrier to collaboration due to longer time frames in research projects contrasting with greater emphasis on quick implementation in industry” [43].

The remaining organizational boundaries that hindered or facilitated IOC were phase specific. During the development phase, boundaries related to organizational cultures [48], intellectual property rights [47], and ideas [48] emerged. These boundaries (potentially) hindered academic-industry [47] or academic-industry-health care-government collaborations [48], as conflicts pushed partners apart or delayed the process. For instance, a small-medium enterprise (SME) had an ongoing discussion with a university regarding the intellectual property rights of a theory applied in the design of eHealth [47]. This discussion delayed the start of the evaluation phase. In the evaluation phase, organizational boundaries related to operational processes hindered researcher-clinician collaboration. For example, IOC was impeded by differences in staff management routines during a randomized clinical trial aimed at evaluating the effectiveness of eHealth for diabetes [46]. In the implementation phase, policy makers became part of the IOC, leading to organizational boundaries between academia, industry, health care, and government [51]. These organizations faced boundaries in values [51] and contexts [51], which increased the complexity of IOC. In addition, differences in convictions regarding future revenues hindered IOC. To illustrate, “The main reason for the struggles experienced in the cooperation among SMEs was that the SMEs differed in their convictions of future revenues because of uncertainties in the financial market and that it was not possible to make a positive business case” [51]. By contrast, smaller organizations appeared to be more flexible, facilitating IOC between academia, industry, health care, social care, and the third sector [49]. In conclusion, each phase contains both general and phase specific organizational boundaries that either facilitate or hinder IOC.

#### Identification

The identification of organizational boundaries facilitated IOC throughout the deployment of eHealth [45,46,48,55]. By identifying these boundaries early, potential problems in IOC that might arise in later stages could be prevented. The authors noted that this identification is ideally carried out in the development phase to ensure that researchers and clinicians are aware of these boundaries during the evaluation phase [46]. Moreover, early identification enhanced the dialogical learning mechanism of reflection, as it prompted dialogue among academic, industry, health care, and governmental organizations facing conflicts caused by organizational boundaries [48]. To stimulate an early identification process, the authors proposed methods to enhance this process. For instance, during the development phase, the principal investigator underwent additional training to better identify organizational boundaries and foster reflection. To illustrate, “To facilitate our interdisciplinary approach, BM=...undertook additional training to familiarize herself with the methods from the different disciplines and consulted experts from other fields before and after each activity” [55]. In addition, previous relationships facilitated academic-industry collaborations. Conversely, working with a new person due to the departure of the previous collaborator hindered academic-industry collaborations [45]. Starting collaborations with established relationships is easier and more efficient because representatives are already familiar with each other’s work styles, unlike when working with someone new [45]. This is well illustrated by the following quote: “Most participants reported they would prefer to keep the same collaborators over time as they learned each other’s work style” [45]. In summary, early identification of organizational boundaries facilitated IOC and was further enhanced through additional training and the establishment of collaborations based on previous relationships.

#### Coordination

The dialogical learning mechanism of coordination facilitated IOC throughout the deployment of eHealth [40-42,45,46,49-52,55]. This coordination depended on the involvement of boundary spanners and the use of boundary objects. Boundary spanners, such as principal investigators, project managers, individuals with diverse expertise, or employees of the Ministry of Health, played a key role in coordinating organizational boundaries, thereby facilitating IOC across the eHealth deployment phases [41,45,52,55]. The specific ways in which these individuals spanned organizational boundaries varied depending on the deployment phase. For instance, in studies that focused on the development phase, the principal investigator coordinated the differing perspectives between health care and industry partners during co-creation sessions. To illustrate, “Throughout the development process, the lead author served as a linking point for all stakeholders and tried to gain and share everyone’s views and opinions” [55]. In studies focusing on the evaluation phase, collaborating with primary care leaders and clinicians from the involved hospital enhanced IOC. These health care professionals could negotiate their involvement and were aware of the organizational boundaries in the operational processes of primary care [41]. Moreover, in the implementation phase, it was not an individual, but rather an organization, that spanned organizational boundaries. A hospital played a key role in facilitating the spread of telehealth by fostering IOC between nearby hospitals [54]. Other authors highlighted the government’s crucial role in facilitating collaboration between developers and owners of a general practice information system [52].

Additionally, boundary spanners utilized boundary objects at the outset and throughout the deployment of eHealth to facilitate IOC. Specifically, the use of boundary objects before the start of any of the 3 deployment phases helped reduce organizational boundaries that could hinder IOC in later phases. This is illustrated by the following quote: “Central objects developed in the initial design phase (collaboration contract, trial protocol, and system components) constitute the necessary foundation for successful collaborative research” [46]. At the start of the eHealth deployment process, boundary objects focused on clarifying the IOC process. For instance, contractual agreements [45,46,49], shared vision statements [51], clear rationales for IOC [42], and role clarity [49] were used. Additionally, throughout the deployment process, specific boundary objects used in any of the 3 phases enhanced IOC. In studies covering the development phase, boundary spanners used boundary objects to facilitate dialogues on the diverse perspectives regarding the design of eHealth [40,46]. One of the methods used to facilitate IOC was the positive brainstorming approach, where individuals were encouraged to communicate with a “Yes, and...” mindset rather than “No, but...” [40]. Another method applied was the participatory design technique, which helped bridge the gap between clinicians and researchers. Experiences from clinicians and researchers collaborating on the introduction of a mobile health (mHealth) system showed that participatory design narrowed their differences [46]. During the evaluation phase, having a trial protocol as a boundary object facilitated collaboration between universities and hospitals [46]. Additionally, during the implementation phase, a business model served as a boundary object that bridged differing visions on future revenues, enhancing IOC [51]. Thus, throughout the eHealth deployment process, boundary spanners and context-specific boundary objects played a crucial role in facilitating IOC.

#### Reflection

Reflection enhanced IOC throughout the development, evaluation, and implementation of eHealth [40,43-45,47,49,51-53]. Specifically, reflecting on values, priorities, goals, visions, and expectations played a key role in facilitating IOC [43-45,47,49]. To illustrate, some authors recommended “Empathize with and explicitly address diverse values and priorities” to facilitate academic-industry collaboration [44]. Others highlighted the facilitating effect of reflective outcomes on academic-industry collaboration or IOC between academia, industry, health care, government, social care, and the third sector. For example, mutual understanding, alignment, and agreement on timelines, goals, and expectations were key factors [43-45,49]. Approaches used to enhance the reflection process during IOC included providing enough time [45] and fostering a culture that unites organizations [40]. Within this culture, it was essential to create space for reflexive learning to enhance mutual understanding between organizations [52]. Additionally, strong relationships and interpersonal dynamics based on trust and mutual respect were crucial for IOC [45]. However, these approaches were less prominent compared with communication [43-45,49]. Communication helped make differences in goals, expectations, values, and priorities explicit, thereby increasing the identification of organizational boundaries in all phases [43]. For example, “outlining and communicating openly about goals and expectations for timeline and priorities” enhanced the academic-industry collaboration [44]. However, IOC was hindered by inauthentic communication [43]. Therefore, communication needed to be open to positively influence IOC [43]. Additionally, communication had to be frequent throughout all phases, with the frequency varying over time. For instance, one study found that the project initiation and onboarding phase required more meetings compared with the project execution phase [45]. In contrast to the dialogical learning mechanisms of identification and coordination, which facilitated IOC differently across deployment phases, the dialogical mechanism of reflection was supported by continuous open communication throughout all phases.

## Discussion

### Principal Findings

**Figure 2:**
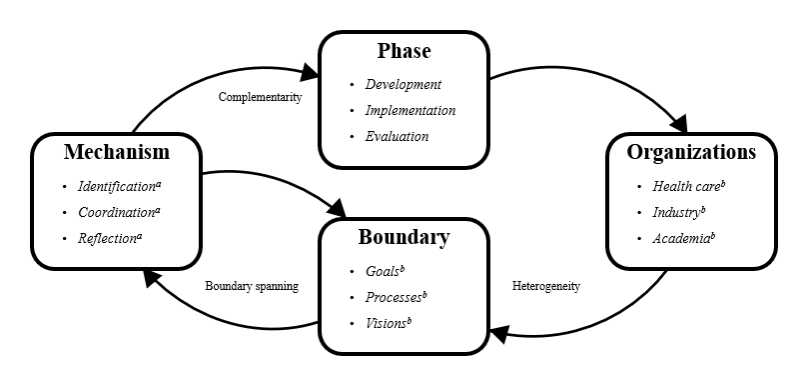
The dynamic process of interorganizational collaboration throughout eHealth deployment. ^a^Dialogical learning mechanisms [25]. ^b^Found in this scoping review.

This scoping review aimed to identify when and how organizational boundaries facilitate or hinder IOC throughout the development, evaluation, and implementation phases of eHealth. The review found that IOC in eHealth deployment is a dynamic process that depends on identification, coordination, and reflection to navigate organizational boundaries. As illustrated in Figure 2, eHealth deployment begins with defining the phase and the organizations involved, leading to organizational boundaries that either hinder or facilitate IOC. The first dialogical learning mechanism for spanning organizational boundaries is identification. This mechanism is enhanced by prior knowledge of boundaries gained through past collaborations or training. The second mechanism, coordination, facilitates IOC by involving boundary spanners and utilizing boundary objects tailored to the specific organizational boundaries that need to be addressed. The third dialogical learning mechanism that helps span organizational boundaries is reflection. To enhance reflection, open and frequent communication is crucial. However, the fourth dialogical learning mechanism, transformation, is not part of the process, as it does not influence IOC in eHealth deployment. In the following paragraphs, the scientific and practical implications of the 3 dialogical learning mechanisms—identification, coordination, and reflection—on IOC during eHealth deployment will be discussed.

### Identification

This review revealed that organizational boundaries hindered or facilitated IOC differently across deployment phases, as each phase required different organizations. The heterogeneity of organizations in eHealth deployment arises from the involvement of diverse entities to optimize eHealth implementation [12]. However, in line with previous literature, this review demonstrated that involving multiple organizations to achieve complementarity also increased the occurrence of organizational boundaries. Thus, involving a variety of organizations is not always advantageous. This can increase the conflicts and tensions caused by organizational boundaries [56,57]. Therefore, awareness of organizational boundaries that may arise from the selection of organizations during the design of eHealth projects is essential. The results of this review contribute to this awareness by identifying, for each deployment phase, which organizations collaborated and how the organizational boundaries between these organizations hindered or facilitated IOC. Additionally, the facilitating effect of the dialogical learning mechanism identification on IOC was revealed. While the importance of identification, involvement, and analysis of the involved organizations [58-61] is acknowledged in the literature, there is limited research adopting a phase specific perspective on how organizational involvement leads to organizational boundaries. These boundaries are predominantly reported in the context of academic-industry collaboration during eHealth deployment. By contrast, organizational boundaries between health care and industry, as well as in collaborations with the government, are not reported. As not all organizational boundaries are reported, there is a limited view of how the involvement of organizations leads to organizational boundaries that facilitate or hinder IOC per deployment phase. In addition, it is difficult to distinguish between general and phase specific organizational boundaries, as well as to assess their relative importance in facilitating or hindering IOC. This underscores the need for further research into how organizational boundaries and dialogical learning mechanisms dynamically occur [62]. After all, only with an understanding of how organizational boundaries arise throughout eHealth deployment is it possible to coordinate boundary spanning and enhance the implementation of eHealth.

### Coordination

The coordination relied on the involvement of boundary spanners and the use of boundary objects. The literature identifies several types of boundary spanners [63] and boundary objects that can span organizational boundaries [64-68]. For example, a boundary spanner can be a ‘representative’ who diffuses knowledge across organizational boundaries [69]. Boundary objects can range from art-based [70] and design methods [71] to software [72] that bridge different social worlds. This scoping review showed that, in addition to the dialogical learning mechanism identification, the dialogical learning mechanism coordination is also phase specific during eHealth deployment. The organizational boundaries within a specific eHealth deployment phase determine the required boundary spanners and boundary objects to enhance boundary spanning and IOC. Therefore, after identifying the organizational boundaries through the identification mechanism, coordination is needed to select boundary spanners and boundary objects that are suited to the specific boundary that must be spanned to enhance IOC. This aligns with prior literature stating that boundary spanners and boundary objects vary by context. Consequently, previous studies have focused on the use, roles, and characteristics of boundary spanners [69,73,74] and boundary objects [75,76] across diverse contexts. However, the effectiveness of methods for identifying, coordinating, and reflecting on boundaries to facilitate IOC before and during an eHealth deployment phase is not reported. For example, the authors of the eligible studies did not assess the effectiveness of the methods, (training) strategies, boundary objects, or the involvement of boundary spanners in facilitating IOC. Without this knowledge, it is difficult to determine which boundary spanners and boundary objects are suitable for spanning boundaries in a specific deployment phase. This insight is urgently needed by individuals involved in IOC who must select appropriate boundary objects and boundary spanners during eHealth deployment. Therefore, further research is essential to enhance the positive impact of coordination on IOC.

### Reflection

Most studies included in this review addressed the importance of the dialogical learning mechanism reflection throughout the deployment of eHealth. Prior literature has presented various forms of reflection serving diverse aims during IOC. For example, in the field of eHealth, the Centre for eHealth Research and Disease Management (CeHReS) roadmap incorporates formative evaluation after each deployment phase [2,12]. These recurring evaluation moments are intended for backward reflection, to assess whether the goals set at the beginning of the deployment phase have been achieved [2]. In addition, the CeHReS roadmap emphasizes forward evaluation to assess the current state of the development process and determine whether improvements are needed to optimize the design and implementation of eHealth [2]. Studies on IOC have highlighted the importance of reflection to establish and reevaluate the roles, tasks, and goals of IOC throughout the process [77]. The findings of this scoping review underscore that reflection on organizational boundaries, and how to span them, for example, through the use of boundary objects and the involvement of boundary spanners, is important in each deployment phase. Specifically, this reflection should occur through open communication to enhance IOC. However, existing frameworks rarely emphasize continuous reflection on organizational boundaries and the application of the 4 dialogical learning mechanisms, instead focusing on the process, design, and implementation aspects of eHealth deployment. Furthermore, while interprofessional communication in health care is well-researched [78,79], there is a lack of studies examining communication across organizational boundaries. In particular, research on how to communicate about organizational boundaries is scarce. For example, few studies explore how frequently communication should occur or how to openly discuss differing goals and values. Additionally, there is a lack of research on how communication contributes to the dialogical learning mechanisms identification and coordination. Therefore, future research is needed to address this knowledge gap on how to communicate during IOC. Moreover, incorporating dedicated moments for reflection is crucial, as the successful deployment of eHealth depends on identifying organizational boundaries and finding effective ways to span them.

### Strengths and Limitations

This scoping review provides a broad overview of organizational boundaries and dialogical learning mechanisms that were not explicitly identified in prior literature. The use of organizational boundaries and the 4 dialogical learning mechanisms of Akkerman and Bakker [25] to analyze the existing literature on barriers and facilitators of IOC was a significant strength, helping to generate this overview. Additionally, incorporating empirical studies, whether they focused on IOC in eHealth deployment or not, provided a more comprehensive understanding. However, findings from different collaboration levels, such as intraorganizational or team-level interactions, as well as other types of boundaries beyond organizational boundaries that can influence IOC, were outside the scope of this review. This limits the practical applicability of the generated overview. Additionally, the eligibility criteria, which restricted the review to English and Dutch articles, may have introduced a language bias. Nevertheless, these limitations present valuable directions for future research to enhance understanding of the complexities of IOC during eHealth deployment. Specifically, more scholarly attention could be given to the occurrence of different types of boundaries at various levels and between different types of organizations.

### Conclusions

IOC in eHealth deployment is a dynamic process that relies on the dialogical learning mechanisms identification, coordination, and reflection to span organizational boundaries. This dynamic nature underscores the need for a phase specific perspective on IOC during eHealth deployment. In each deployment phase, the organizational boundaries that arise from the selection of organizations must be identified. Additionally, each phase requires coordination to determine which boundary spanners and boundary objects can effectively span these organizational boundaries. Furthermore, in each phase, reflection, established through open and frequent communication, can enhance IOC. This scoping review is the first to present a phase specific view of the influence of organizational boundaries and dialogical learning mechanisms during eHealth deployment. However, future research is needed on organizational boundaries and the use of dialogical learning mechanisms in each deployment phase to better understand the complexities of collaborating across organizational boundaries throughout eHealth deployment.

## Appendix

Multimedia Appendix 1 PRISMA-ScR (Preferred Reporting Items for Systematic Reviews and Meta-Analyses extension for Scoping Reviews) checklist.

Multimedia Appendix 2 Search strings.

## Abbreviations

CeHReS: Centre for eHealth Research and Disease Management
IOC: interorganizational collaboration
mHealth: mobile health
PRISMA-ScR: Preferred Reporting Items for Systematic Reviews and Meta-Analyses Extension for Scoping Reviews
SME: small-medium enterprise
