# Supplementary material for: Collaborating Across Organizational Boundaries to Develop, Evaluate, and Implement eHealth: Scoping Review
Source: J Med Internet Res. 2025 Jun 10;27:e67839. doi: 10.2196/67839 (PMC12188139; doi:10.2196/67839)
Supplement: Multimedia Appendix 2 [file jmir_v27i1e67839_app2.pdf]

Multimedia appendix 2 search strings

Database	Search string
Web of Science	((AB=(interorgani* OR inter-organi* OR alliance OR consorti* OR network* OR inter-firm OR interfirm OR "joint venture" OR joint-venture OR coalition OR organization* OR organisation* OR firm* OR compan* OR stakeholder* OR public OR private OR academic OR industr* OR universit*)) AND AB=(collaborat* OR cooperat* OR relationship* OR partnership*)) AND AB=("digital health" OR "electronic health" OR "mobile health" OR "web-based health" OR eHealth OR e-Health OR mHealth OR m-health OR telemedicine OR tele-medicine OR telehealth OR tele-health OR "eMental health" OR "e-Mental health")
PubMed	((interorgani*[Title/Abstract] OR inter-organi*[Title/Abstract] OR alliance[Title/Abstract] OR consorti*[Title/Abstract] OR network*[Title/Abstract] OR inter-firm[Title/Abstract] OR interfirm[Title/Abstract] OR "joint venture"[Title/Abstract] OR joint-venture[Title/Abstract] OR coalition[Title/Abstract] OR organization*[Title/Abstract] OR organisation*[Title/Abstract] OR firm*[Title/Abstract] OR compan*[Title/Abstract] OR stakeholder*[Title/Abstract] OR public[Title/Abstract] OR private[Title/Abstract] OR academic[Title/Abstract] OR industr*[Title/Abstract] OR universit*[Title/Abstract]) AND (collaborat*[Title/Abstract] OR cooperat*[Title/Abstract] OR relationship*[Title/Abstract] OR partnership*[Title/Abstract])) AND ("digital health"[Title/Abstract] OR "electronic health"[Title/Abstract] OR "mobile health"[Title/Abstract] OR "web-based health"[Title/Abstract] OR eHealth[Title/Abstract] OR e-Health[Title/Abstract] OR mHealth[Title/Abstract] OR m-health[Title/Abstract] OR telemedicine[Title/Abstract] OR tele-medicine[Title/Abstract] OR telehealth[Title/Abstract] OR tele-health[Title/Abstract] OR "eMental health"[Title/Abstract] OR "e-Mental health"[Title/Abstract])
CINAHL	AB (interorgani* OR inter-organi* OR alliance OR consorti* OR network* OR inter-firm OR interfirm OR "joint venture" OR joint-venture OR coalition OR organization* OR organisation* OR firm* OR compan* OR stakeholder* OR public OR private OR academic OR industr* OR universit*) AND AB (collaborat* OR cooperat* OR relationship* OR partnership*) AND AB ("digital health" OR "electronic health" OR "mobile health" OR "web-based health" OR eHealth OR e-Health OR mHealth OR m-health OR telemedicine OR tele-medicine OR telehealth OR tele-health OR "eMental health" OR "e-Mental health")
PsycINFO	AB (interorgani* OR inter-organi* OR alliance OR consorti* OR network* OR inter-firm OR interfirm OR "joint venture" OR joint-venture OR coalition OR organization* OR organisation* OR firm* OR compan* OR stakeholder* OR public OR private OR academic OR industr* OR universit*) AND AB (collaborat* OR cooperat* OR relationship* OR partnership*) AND AB ("digital health" OR "electronic health" OR "mobile health" OR "web-based health" OR eHealth OR e-Health OR mHealth OR m-health OR telemedicine OR tele-medicine OR telehealth OR tele-health OR "eMental health" OR "e-Mental health")
